# Multimodal inverse kinematics significantly improves IMU-based biomechanical analyses

**DOI:** 10.1038/s41598-025-33021-7

**Published:** 2025-12-23

**Authors:** Iris Wechsler, Julian Shanbhag, Niklas Schlechtweg, Martin Vossiek, Anne D. Koelewijn, Sandro Wartzack, Jörg Miehling

**Affiliations:** 1 Engineering Design, Department of Mechanical Engineering, Friedrich-Alexander-Universität Erlangen-Nürnberg, 91058 Erlangen, Germany; 2 Institute of Microwaves and Photonics, Department of Electrical Engineering, Friedrich-Alexander-Universität Erlangen-Nürnberg, 91058 Erlangen, Germany; 3 Chair of Autonomous Systems and Mechatronics, Department of Electrical Engineering, Friedrich-Alexander-Universität Erlangen-Nürnberg, 91052 Erlangen, Germany

**Keywords:** Inverse kinematics, Multimodal motion data, Biomechanical modeling and simulation, Tracking methods, Engineering, Mathematics and computing

## Abstract

In musculoskeletal simulations, IMU-based approaches are often compromised by errors such as joint angle drift and offset errors due to calibration inaccuracies. These errors can compromise the accuracy of both kinematic and dynamic outcomes. This study presents a simulation-based investigation that uses synthetic inertial and positional data to systematically assess the potential of integrating spatial reference information into IMU-driven inverse kinematics analyses. Optical motion capture data was captured and error-free kinematic and dynamic data was created based on the optical motion capture data. The error-free data was then used as a reference. Based on this reference data, synthetic orientation and position data was created, incorporating a range of error types and magnitudes (e.g., sensor noise, drift, misalignment). To create the IMU-based analysis results, we calculated relative quaternions based on the orientation data which were then converted into Euler angles. We then conducted a sensitivity analysis to determine the spatial accuracy required in the position data to effectively compensate for typical IMU errors. Across all error types and magnitudes, the multimodal inverse approach (using both synthetic IMU and positional data) yielded significantly more accurate results than solely IMU-based analyses. Specifically, the mean joint angle RMSE decreased by ${63}{\%} (\sim {5}^{\circ })$, the mean joint torque RMSE by ${80}{\%} (\sim {17}\,\hbox {Nm})$, the mean residual force RMSE by ${25}{\%} (\sim {9}\,\hbox {N})$, and the mean residual torque RMSE by ${70}{\%} (\sim {26}\,\hbox {Nm})$. Future research will evaluate the effectiveness of the multimodal inverse kinematics approach when applied to real-world measurement data.

## Introduction

Musculoskeletal simulations are used to estimate biomechanical variables that cannot (easily) be measured in reality, such as joint angle and joint velocity data during movement, or the muscle forces required to perform these movements. Simulations are used in a variety of fields to answer a range of (research) questions. For instance, in the field of medical technology they are used to assess the outcome of anterior cruciate ligament surgery^1,2^, in ergonomics, to assess a worker’s workload^3^, and in sports science to optimize an athlete’s performance^4^. To analyze the motion data, it must first be captured. The most common approach to measure human motion data is optical motion capture. Small reflective markers are attached to specific points on the skin (usually on anatomical landmarks), and the trajectories of these markers are then measured using a set of high-resolution cameras while the participant moves within a calibrated measuring space. There are also alternative technologies available. Inertial measurement units (IMUs) are a frequently used alternative^5–9^. One of the key advantages of IMU-systems is that they do not require a calibrated capture volume, which allows users to record motion data more flexibly, e.g. in outdoor environments^10^. Further, completely non-invasive and markerless approaches, meaning no markers or sensors have to be attached to the participants, have been developed that use either RGB cameras^11–13^, depth cameras^14–16^ or a combination of both to record human movement^17^. These approaches are a simple and cost-effective way of recording movement as no expensive measurement system is required. The most recent advancement are radar-based motion capture approaches^18,19^. Although using radars for motion tracking is still in its early stages of development, results so far are promising. This is due to the fact that radars are not affected by lighting conditions and are capable of directly measuring speeds, distances and angles^18^.

Following the motion capture, the data is transferred to a musculoskeletal model. Musculoskeletal models are multibody systems that depict a person in the virtual world. The models consist of rigid bodies, joints and actuators, which respectively represent the bones, joints and muscles of the human body. A conventional approach to compute joint kinematics (joint angles, velocities and accelerations) and joint torques or muscle forces from a recorded motion is the inverse approach. It consists of inverse kinematics, followed by inverse dynamics and static or dynamic optimization. However, musculoskeletal simulations are simplifications of reality, thus the simulation results are error-prone. For the inverse approach, residual forces and torques (’residuals’) appear at the segment connected to the ground frame which is often the pelvis of a biomechanical model. Residuals have no physical cause and therefore are often called ’hand of god’ forces, making the simulation results physically inconsistent^20,21^, as residual forces are not needed in reality. Therefore, deviations between reality (represented by the experimental data) and the simulation remain. We called this deviation the sim2real gap. In a previous study we identified and analyzed approaches how to handle this gap^22^. In short, both the level of detail of the musculoskeletal models as well as the tracking method used, affect the size of the sim2real gap. We decided to focus on and analyze available tracking methods in the review. Based on the identified and clustered results we concluded that one possible solution to handle the sim2real gap may be tracking multimodal sensor data, as multimodal approaches led to smaller deviations between model and measurement data (e.g. marker deviations or IMU-based body orientations). Pearl et al.^23^ used trajectory optimization to fuse RGB camera and IMU data. The authors reported that the multimodal solution outperformed single-modality approaches using either IMU or video camera data alone. Additionally, trajectory optimization, alone^24^ or combined with machine learning approaches^25^, has been successfully used to compensate for or prevent IMU drift by using raw IMU data directly, avoiding sensor data integration and associated drift. However, applications have focused solely on two-dimensional gait and running movements thus far. Mallat et al.^26^ used a Kalman filter to fuse RGB camera data and IMU sensor data. The authors reported reduced motion tracking errors as the sensor technologies compensated each other’s weaknesses (marker occlusion and IMU sensor drift respectively). In contrast, Atrsaei et al.^27^ fused depth camera data and IMU sensor data for human motion tracking, also using a Kalman filter. The sensor fusion led to smaller deviations between computed joint angles and reference data calculated from marker-based motion data.

Even though Kalman filters have been successfully used for musculoskeletal simulations, they are not without limitation. The quality of tracking results computed using a Kalman filter are significantly influenced by the quality of chosen parameters e.g. covariances of the system and measurement noise. Further, specific assumptions are often taken to enhance convergence, including constant acceleration or jerk^28,29^, periodicity of motions^26^ or bilateral symmetry^26^. The conventional inverse kinematics approach does not rely on such assumptions. Joint angles are calculated by minimizing the difference between experimental and model data (either marker positions or orientations) for each frame. However, a marker-based inverse kinematics approach requires a dedicated motion capturing lab as well as attaching the markers on the skin of a participant ideally, making the capturing inflexible, cumbersome, and invasive for the participants. For IMU-based motion data, the capture process is more flexible as well as user-friendly, as the sensors are mostly attached to the body using flexible bands which can be placed above the clothing of a participant. One disadvantage of IMU-based motion data is, that the data may be affected by various errors such as sensor drift^30^ and calibration errors^31^, leading to inaccurate analysis results. Therefore, the approaches are not suited for use cases requiring high levels of accuracy and reliability, such as within a clinical context. A combination of both position and orientation data within the framework of the inverse kinematics approach should lead to more accurate biomechanical analysis results, as both data types complement each other. In the context of a simulation study, we want to investigate whether single position references, determined by alternative motion capture systems such as RGB cameras, depth cameras or radars, enhance the accuracy of IMU-based inverse kinematics results. The intention is thus not to use a complete set of highly precise marker position data that has been recorded by an optical measurement system. Instead, the focus is on investigating the impact of individual, less precise position data (one per segment).

The objective of this simulation study was to investigate the effect of adding position data of single references to IMU-based joint angle data within the framework of an inverse kinematics analysis. We investigated the effect on the calculated kinematics (joint angles) and dynamics (joint torques and residuals) based on the multimodal approach. As part of a sensitivity study, we wanted to find out the extent of error in the IMU-based joint angle data that can be compensated for by position data of varying accuracy. The approach is implemented in OpenSim – a framework for musculoskeletal simulations^32^. We created synthetic sensor data based on a reference simulation generated based on marker-based motion capturing. We investigated the effect of different errors and error sizes for both the IMU-based orientation data and position data on the resulting kinematics and dynamics.

## Methods

### Model description

In this study, we used the Rajagopal lower limb model^33^. It consists of 20 bodies and has 37 degrees of freedom. Only experimental data for the lower body was captured. Consequently, the degrees of freedom of the upper body were locked. In addition, the metatarsophalangeal joint on each foot was locked. Therefore, the model had 16 free degrees of freedom in total. Virtual IMU sensors were placed on the pelvis, the upper and lower leg and the foot on each side. In addition to the markers corresponding to the experimental marker set, seven markers were placed at the origin of the virtual IMU sensor coordinate systems. To avoid uncertainties introduced by muscle modeling, muscles were removed and replaced with ideal torque actuators for each degree of freedom. This enabled us to assess the separate impact of the tracking method on joint kinematics and inverse dynamics results. In addition, point and torque actuators for each coordinate (x,y,z) were added to the origin of the left and right toes of the model to generate synthetic ground reaction forces using the reference data.

### Experimental data collection and data pre-processing

Gait data was captured for one participant (female, 28 years old, ${1.6}\,\hbox {m}, {56}\,\hbox {kg}$) walking on a treadmill using the marker-based motion capture system OptiTrack^34^. The motion capture procedure was initiated with the participant standing on the treadmill and continued for a duration of 30 seconds with a self-chosen walking speed. We analyzed the first ten seconds of motion encompassing the transition from standing to walking. The Rizzoli lower body marker set^35^ comprising 22 markers was used in the motion capture process. Overall, 12 cameras (type: Flex 13) were used for capturing the passive markers (diameter: 14 mm). In addition to the collection of gait data, motion data of a static pose was captured for the purpose of model scaling. The motion data was captured with a measuring frequency of ${120}\,\hbox {Hz}$. An auto-labeling function was used to label the data. Unidentified or misidentified markers were manually labeled. We encountered problems with some markers due to marker occlusion. Therefore, we used interpolation methods to recalculate the missing data. The marker data was filtered using a third order Butterworth Filter with a cutoff frequency of ${6}\,\hbox {Hz}$. The study was conducted in accordance with the Declaration of Helsinki and approved by the Institutional Review Board (or Ethics Committee) of Friedrich-Alexander-Universität Erlangen-Nürnberg (protocol code 20-489_1-B). The participant provided written informed consent prior to inclusion in the study.

### Creation of reference data

The lower body of the generic Rajagopal model was scaled based on marker data from the static pose. Using the OpenSim scaling tool, all 22 markers of the Rizzoli lower body marker set were used to scale the model. Using inverse kinematics, the reference kinematics data was computed. The computed results were filtered using a third-order Butterworth filter with a cutoff frequency set at ${6}\,\hbox {Hz}$. Based on the calculated generalized coordinates, static optimization was used to compute reference joint torques. Ground reaction forces were not measured using a force plate, but were instead estimated using the model and the reference generalized coordinates. Synthetic ground reaction forces were computed using the point and torque actuators added to the toes. To generate realistic ground reaction forces, the point and torque actuators on each foot were only activated when the respective foot was in contact with the ground. Heel strike and toe off time frames were estimated by analyzing the processed marker data. Heel strike time frames were determined through the identification of frames in which the velocity of the heel marker was found to be zero. The identification of toe-off time frames was achieved by the identification of peaks in the vertical acceleration data of the toe marker. As the reference data was used to calculate the synthetic ground reaction forces, the reference inverse dynamics results were free of residuals.

### Creation of synthetic data

Based on the reference kinematics data, the synthetic position and orientation data for the sensitivity analysis was created. To create the noise-free position data, the position of each of the seven markers placed in the virtual IMU sensors was extracted for every frame. To generate noisy position data, two errors were added to the noise-free reference position data: a white Gaussian noise ($X \sim {\mathscr {N}}(\mu , \sigma ^2)$) depicting measurement noise and a marker offset depicting calibration errors. For both errors, three sizes of errors were chosen, see Table 1. The types and sizes of error for the position data were selected in reference to data accuracy for the determination of sensor positions using radars, as outlined in^36^. Furthermore, the position data has been adjusted to include dropout rates (missing values). For each position reference, a random dropout rate of between ${20}\%\,\hbox {to}\,{40}{\%}$ has been implemented. The missing values were then filled using cubic splines. To generate synthetic orientation data that imitates experimental IMU-based orientation data, angular velocities were calculated using the virtual IMU-sensors. Four different error types were added to each component of the angular velocity vector: a bias, a misalignment error, a bias drift and a Gaussian noise defined by a noise density value. The bias and the misalignment error did not change in size. For the bias drift and Gaussian error, three levels of errors were chosen. Bias and misalignment were kept constant as they represent time-invariant, device-specific errors for a given setup. In contrast, bias drift and noise density were varied to simulate time-dependent effects and different sensor quality levels, respectively, enabling a broader evaluation of the robustness of the multimodal inverse kinematics approach. The types and sizes of errors were chosen with reference to the error values of the gyro sensor built into the ICM-20689 motion tracker, as documented in the technical datasheet^37^. The noisy angular velocity data was then integrated to generate noisy orientation data. In addition to the four errors added directly to the angular velocities, a random but constant misalignment error between ${5}^{\circ }\,\hbox {to}\,{10}^{\circ }$ was added to each sensor data to model the effect of incorrect sensor placement on the body. To model the effect of magnetic disturbance, a yaw drift error of ${0.01}\,\hbox {rad/s}$ was added to the integrated angular velocities. The orientation data was expressed as quaternions. The size of the errors for every level is listed in Table 1. By combining all position and orientation error levels, 81 pairs of input files were created in total.

**Table 1:** Error sizes for all types of errors investigated in the sensitivity analysis.

	Marker offset	Marker noise	Bias drift	Gyro noise density	Bias	Misalignment	Yaw drift
Small	[0.001, 0.001, 0.0015]	0.005	1e−5	0.001	0.0055	1%	0.01
Medium	[0.005, 0.005, 0.025]	0.01	5e−4	0.005	0.0055	1%	0.01
High	[0.01, 0.01, 0.035]	0.05	2e−3	0.01	0.0055	1%	0.01

Marker Offset and Marker Noise in $m$, Bias drift in $rad/\sqrt{s}$, Gyro noise density in $rad/s/\sqrt{Hz}$, Bias in $rad/s$ and Yaw drift in $rad/s$.

### Creation of IMU-based motion data

The synthetic IMU-based motion data was created based on the synthetic orientation data. Joint angles were calculated based on the synthetic orientation data expressed as quaternions. The hip, knee and ankle angles were calculated by computing the relative quaternion between two quaternions expressing the orientation of adjacent segments. The relative quaternion is expressed in axis-angle form. For each joint angle, the axis angle form was then transformed to Euler angles corresponding to the degrees of freedom of the biomechanical model. As we generated orientation data based on the virtual gyro data, only data for the rotational degrees of freedom of the model were generated for the IMU-based solution. The computed joint angles were filtered using a third-order Butterworth filter with a cutoff frequency set at ${6}\,\hbox {Hz}$. No data was generated for the translational generalized coordinates (x-, y-, z-translation of the pelvis). Instead, fixed values were chosen to hold the model in place.

### Multimodal inverse kinematics

The multimodal inverse kinematics approach used both position and joint angle data as input. Consequently, the IMU-based motion data described in the preceding paragraph is used. Analogous to the marker-based inverse kinematics approach, it solved a least-squares optimization problem for every timestep. However, in addition to minimizing model–marker deviations at each time step, deviations between model and experimental joint angles are also minimized. The multimodal inverse kinematics results are calculated by solving the following least squares problem for each timestep:

1 $$\begin{aligned} \min J = \sum _{i=1}^7 (w_{mi} \cdot (m_i - m_{di})^\top (m_i - m_{di})) + \sum _{j=1}^{13} (w_{qj} \cdot (q_j - q_{dj})^2) \end{aligned}$$

where $m_i$ and $m_{di}$ are the model and desired marker positions, $q_j$ and $q_{dj}$ are the model and desired generalized joint coordinates and $w_{mi}$ and $w_{qj}$ are the marker and generalized coordinates weightings. For the spatial input data, only the markers positioned at the origin of the virtual IMU-sensors were used. For the rotational input data, joint coordinate values for all free degrees of freedom of the model were used. By using both spatial and rotational data as input, joint coordinate values are computed for all translational and rotational degrees of freedom.

A systematic approach was employed to determine appropriate weightings for the marker and generalized coordinate data. Initially, the error-free reference dataset was used to compute the variance of both data types, enabling a variance-based normalization of the weighting factors. The global variance across all marker positions ($\sigma ^2_{m} = 0.1004$) and generalized coordinates ($\sigma ^2_{q} = 0.1019$) was calculated and the weighting factors were then determined accordingly ($w_{mi} = 9.81$ and $w_{qi} = 9.96$), see Equation 2 and 3 (with $\gamma = 1$). Kinematic results were then computed for all 81 analyses. Analysis of the resulting mean trajectories from both the IMU-based and the multimodal approaches revealed that the marker data had little to no influence on the multimodal inverse kinematics outcomes when using variance-based weightings. Consequently, a focused grid search was conducted to identify more effective weighting parameters. In this process, the weighting of the joint angle data was systematically reduced to evaluate the relative contribution of the marker information.

2 $$\begin{aligned} w_{mi} = \frac{1}{\sigma ^2_{m}} \end{aligned}$$

3 $$\begin{aligned} w_{qj} = \gamma \cdot \frac{1}{\sigma ^2_{q}}, \text { with } \gamma \in \{ 10^i | -8 \le i \le 0 \} \end{aligned}$$

**Figure 1:**
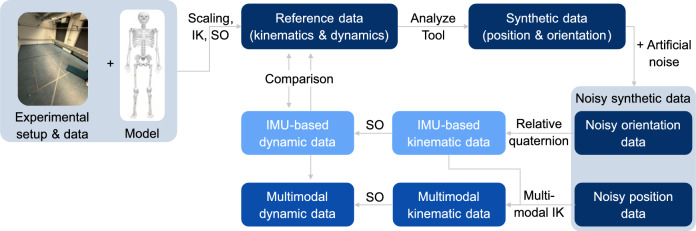
Overview of the simulation study. Reference data was generated using marker-based motion capture and a biomechanical model. Synthetic sensor data was then created from this reference. The IMU-based and multimodal inverse kinematics approaches were applied to analyze the data, and their results were compared to the reference to assess performance.

To determine the optimal weighting configuration, kinematic data for all 81 datasets were computed for each tested weighting pair. The mean joint angle root mean square error (RMSE) between the reference and multimodal results was used as the evaluation metric to identify the most suitable weightings. The resulting mean RMSE values for all tested combinations are provided online as Supplementary Table S1. Based on this evaluation, final weightings of 9.81 for the marker data and $9.96 \times 10^{-6}$ for the generalized coordinates were selected. The computed generalized coordinates were filtered using a third-order Butterworth filter with a cutoff frequency set at ${6}\,\hbox {Hz}$. A range of pelvis marker weightings was investigated to determine whether different weightings of the spatial reference for the base segment affect the size of residual forces and torques. The original weighting was adjusted using a scaling parameter $\epsilon$, with $\epsilon \in \{ 0.01, 0.1, 10, 100 \}$. All other weightings remained the same. The different steps of the simulation study are illustrated in Fig. 1.

### Statistical analysis

To assess whether the inclusion of single position data improves IMU-based kinematic results in the context of this simulation study, paired t-tests were conducted to compare the RMSE values of joint angles and joint torques between the IMU-based and multimodal analysis results, using the reference data as a benchmark. For statistical analysis of dynamic data, residual torques (pelvis rotations) were combined with joint torques. As no data was generated for the IMU-based solution for pelvic translations, no statistical analysis was performed on this data. To evaluate the effect of error types and sizes on the RMSEs, the t-tests were calculated on grouped data based on error type and size. As the orientation data-based method is not affected by the error types of the position data, paired t-tests were only calculated for both orientation errors. Therefore, to compare the joint angle and joint torque data, 6 paired t-tests were calculated. To avoid an inflated Type 1 error rate, we used the Bonferroni-Holm correction to adjust the alpha value for each t-test. The adjusted alpha values were calculated using the following equation:

4 $$\begin{aligned} \alpha _{\text {Holm},i} = \frac{\alpha }{m - i + 1} \end{aligned}$$

where $\alpha$ is the original significance level (0.05), *m* is the number of tests performed (6), and *i* is the rank of the test in order of p-values. 95% Confidence Intervals (CIs) were calculated on the mean difference between RMSEs for each group to further analyze the results. Mean differences were calculated by subtracting the RMSE for the IMU-based approach of the RMSE of the multimodal approach. CIs not including the zero value then indicate a significant mean difference between RMSEs. Before performing the t-test, all groups of data were tested for normality using the Shapiro-Wilk test.

In order to further analyze the impact of position-based error types on the quality of multimodal approach outcomes, the RMSEs for the multimodal approach are analyzed depending on orientation data-based errors and grouped by spatial error sizes. This enables the analysis of the influence of position data accuracy on the effect of orientation errors. To facilitate the analysis, a mean position error was calculated. For each marker offset level (low, medium, high), the mean value is calculated across all marker noise levels for each orientation level. This calculation is perfomed for both orientation errors.

## Results

### Comparison of kinematic data

**Figure 2:**
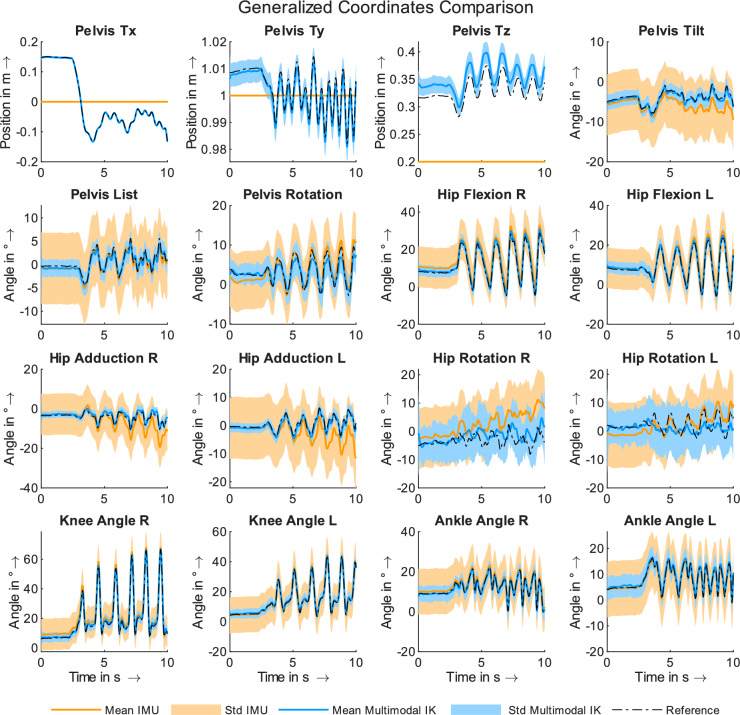
Mean generalized coordinate curves and their standard deviation calculated on the basis of 81 analyses for the IMU-based and multimodal inverse kinematics solution.

We analyzed the computed generalized coordinates for the IMU-based and the multimodal inverse kinematics approach to determine whether the addition of single position references enhances the kinematic results of the IMU-based solutions. Figure 2 shows mean generalized coordinate values as well as standard deviations calculated for 81 analyses for all actuated degrees of freedom for both inverse kinematics analyses results. The reference data is depicted for comparison. In general, the mean curves for all joint angles correspond well to the reference curves for both approaches. However, a better match can be observed for the multimodal inverse kinematics solution. In addition, the standard deviations are larger for the IMU-based solution across all joint angles, with the exception of the hip rotation angles. With regard to the hip rotation and hip adduction angles, a drift in the data can be observed in the IMU-based solutions. With regard to the multimodal approach, such a drift is not observable. For the pelvis translations, a good match can be observed between the multimodal approach and the reference data for the x- and y-translation. For the z-coordinate, a steady offset can be observed. As we used fixed data for the IMU-based approach, large deviations can be observed. Table 2 lists the mean joint angle RMSE values for both approaches with regard to the reference data. The mean RMSE for the IMU-based solution was more than twice that of the multimodal approach. Mean and standard deviation RMSE values for each degree of freedom can be found online as Supplementary Table S2. Incorporating position data enhances the accuracy of computed kinematics, as reflected in both qualitative and quantitative analyses.

**Table 2:** Mean joint angle ($^{\circ }$), mean joint torque ($\hbox {Nm}$) RMSEs and mean residual force ($\hbox {N}$) and torque ($\hbox {Nm}$) RMSEs for both approaches in relation to the reference data.

	IMU	Multimodal
Joint angle RMSE	8.83	3.25
Joint torque RMSE	21.95	4.70
Mean residual forces	34.93	26.15
Mean residual torques	36.59	10.80

Mean residual force and torque RMSE values were computed by averaging the mean values across the x-, y-, and z-directions for all analyses.

**Figure 3:**
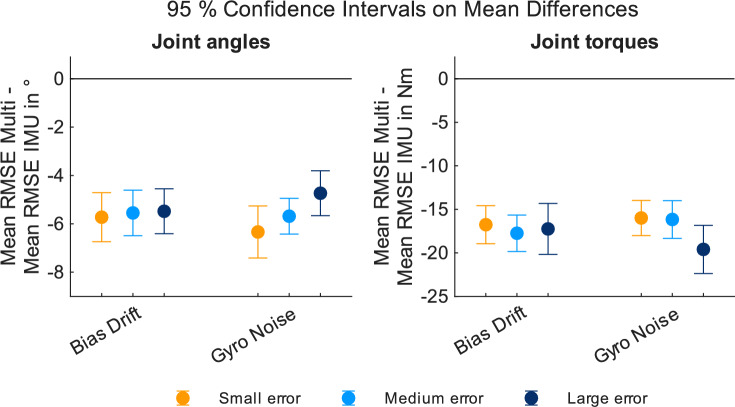
95% CIs on mean differences between RMSE values for IMU-based and multimodal joint angle (left) and joint torque (right) values in relation to the reference data. CIs not including zero value indicate significant difference between RMSE values.

Next, we analyzed the mean difference between the joint angle RMSE values for both approaches to determine whether the addition of position data significantly enhances the computed kinematics. Normality was tested using the Shapiro–Wilk test and was not violated in any of the 6 groups ($p \, > \,0.05$ for all). The calculated paired t-tests showed significant differences for both groups and for all levels of error. Figure 3 shows the ${95}{\%}$ CIs for the mean differences between both approaches. For all orentation-based error types and levels of errors, the multimodal approach resulted in significantly smaller RMSEs as indicated by the negative values of the ${95}{\%}$ CIs. The size of the mean differences was consistent ($\sim {5^{\circ }}\,\hbox {to}\,{6^{\circ }}$) across the errors and levels of errors. An overview of the statistical analysis, showing mean differences, upper and lower bounds of the ${95}{\%}$ CIs, and p-values obtained from the t-tests can be found online as Supplementary Table S3.

Figure 5 shows the joint angle RMSEs of the multimodal approach depending on the orientation errors and grouped by the size of the position errors. The orientation errors have a limited impact on joint angle RMSEs, as demonstrated by the parallel and non-intersecting lines in the RMSE curves for bias drift and gyroscope noise erros. Furthermore, the level offset resulting from position errors remains relatively consistent across all levels of orientation error. However, a clear offset can be seen for the different levels of position error.

### Comparison of dynamic data

**Figure 4:**
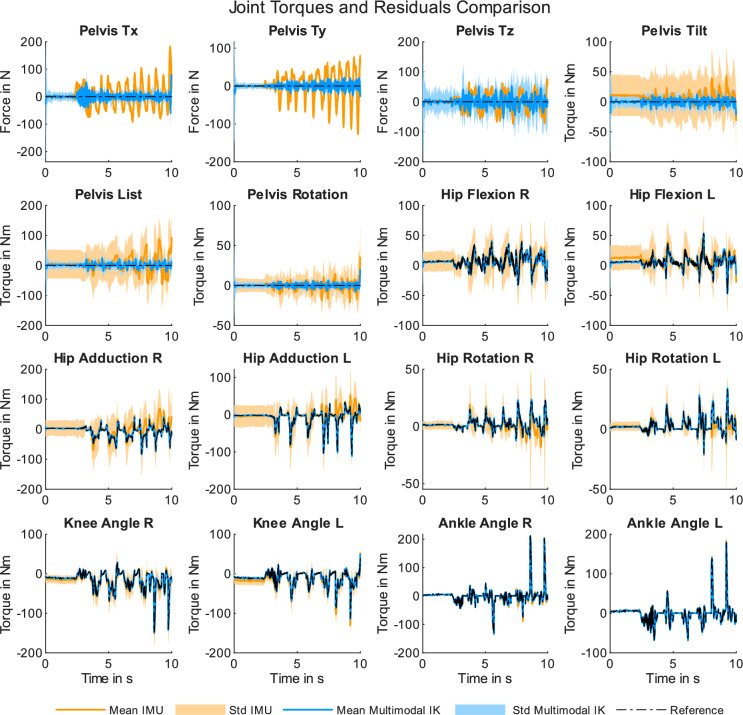
Mean joint torque and residuals curves and their standard deviation calculated on the basis of 81 simulations for the IMU-based and multimodal inverse kinematics solution.

We analyzed the computed torques and residuals for the IMU-based and the multimodal inverse kinematics approach to determine whether the addition of single position references enhances the dynamic results of the IMU-based solutions. Figure 4 shows mean joint torques as well as standard deviations calculated for 81 analyses for all actuated degrees of freedoms for both approaches. In addition, mean residual forces and torques as well as their standard deviations are depicted. In general, computed joint torques correspond well to the reference values for both approaches. However, a better match can be observed for the multimodal solution. The standard deviation values are larger for the IMU-based solution. The mean residual torque values ranged between ${-63.94}\,\hbox {Nm}$ and ${95.19}\,\hbox {Nm}$ for the IMU-based solution and ${-22.29}\,\hbox {Nm}$ and ${25.34}\,\hbox {Nm}$ for the multimodal approach. Mean residual forces ranged between ${-127.03}\,\hbox {N}$ and ${183.66}\,\hbox {N}$ for the IMU-based solution and ${-60.01}\,\hbox {N}$ and ${78.40}\,\hbox {N}$ for the multimodal approach. Table 2 lists the mean joint torque RMSE values for both approaches with regard to the reference data. For the IMU-based solution, the mean RMSE was about five times higher compared to the multimodal approach. In addition, mean residual force and torque RMSE values were smaller for the multimodal approach compared to the IMU-based solution. Mean and standard deviation RMSE values for each degree of freedom can be found online as Supplementary Table S2. The mean residual force and torque RMSE values for the swing and stance phase, per coordinate component, and per approach are listed in Table 3. The multimodal approach resulted in consistently smaller values in both phases and all coordinates. Different pelvis marker weightings did not positively affect residual force and torque RMSEs. While increasing the weighting by a factor of 10 produced consistent results, further values of the scaling factor $\epsilon$ led to higher residual RMSE values. Exact values for all investigated pelvis marker weightings can be found online as Supplementary Table S5. Incorporating position data enhances the accuracy of computed dynamics, as reflected in both qualitative and quantitative analyses.

**Table 3:** Residual forces and torques RMSE values for both approaches during stance and swing phase of gait, for each coordinate component.

	RMSE						Multimodal					
	Force			Torque			Force			Torque		
	x	y	z	x	y	z	x	y	z	x	y	z
Stance Phase	40.23	36.86	18.64	12.69	19.35	4.41	13.83	5.80	10.24	4.30	4.40	2.00
Swing Phase	43.93	32.24	32.01	8.21	27.98	4.61	9.66	7.02	18.40	4.14	8.45	2.13

We analyzed the mean difference between the joint torque RMSE values for both approaches to determine, whether the addition of position data significantly enhances the computed dynamics. Normality was tested using the Shapiro–Wilk test and was not violated in any of the 6 groups ($p \, > \,0.05$ for all) The calculated paired t-tests showed significant differences for all groups and for all levels of error. Figure 3 shows the ${95}{\%}$ CIs for the mean differences between both approaches. For all orientation-based error types and levels of errors, multimodal inverse kinematics based results resulted in significantly better dynamic results (compared to the reference data) as well as to smaller residuals. The mean difference was consistent ($15~\text {Nm}-20~\text {Nm}$) across all error sizes for both errors. An overview of the statistical analysis, showing mean differences, upper and lower bounds of the ${95}{\%}$ CIs, and p-values obtained from the t-tests can be found online as Supplementary Table S4.

**Figure 5:**
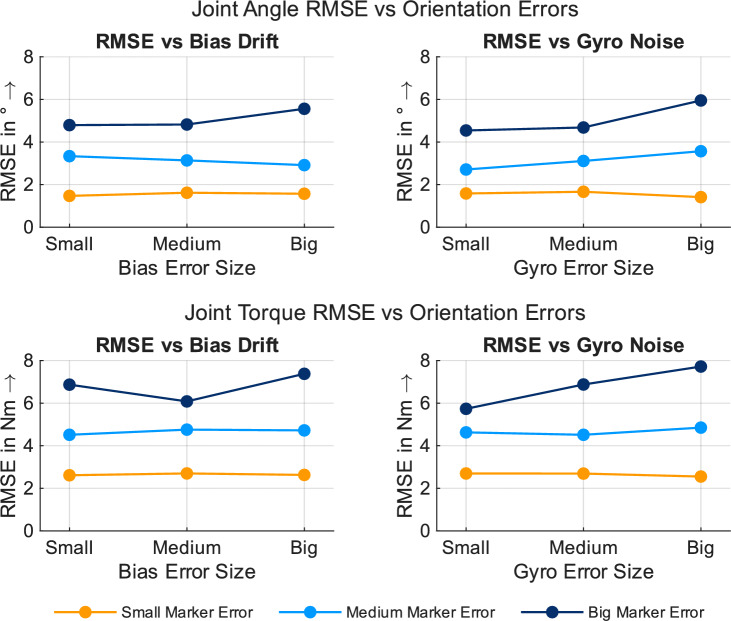
Joint angle (top) and joint torque (bottom) RMSE values for the multimodal approach depending on orientation-based errors and grouped by the size of spatial errors.

Figure 5 shows the joint torque RMSEs of the multimodal approach depending on the orientation errors and grouped by the size of the position errors. The orientation errors have a limited impact on the joint torque RMSEs, as demonstrated by the parallel and non-intersecting lines in the RMSE curves for bias drift and gyroscope noise errors. Furthermore, the level offset resulting from position errors remains relatively consistent across all levels of orientation error. However, a clear offset can be seen for different levels of position error.

## Discussion

In this simulation study, we introduced a multimodal inverse kinematics approach to improve IMU-based motion data by incorporating single position reference data. We performed a sensitivity analysis using synthetic data to assess how much error in IMU-derived joint angle data can be offset by position data with varying accuracy levels. Two types of errors, each with three levels of severity, were introduced into the position and orientation data. In total, 81 pairs of input files and analyses were conducted. Across all error types and magnitudes, the multimodal inverse approach produced notably more accurate results compared to the reference data. For both kinematic (joint angle) and dynamic (joint torque and residuals) analysis results, this approach led to a substantial reduction in RMSEs. Specifically, the mean joint angle RMSE decreased by ${63}{\%}$, the mean joint torque RMSE decreased by ${80}{\%}$, the mean residual force RMSE was reduced by ${25}{\%}$, and the mean residual torque RMSE was reduced by ${70}{\%}$.

While our proposed multimodal approach yielded significantly enhanced kinematic and dynamic analysis results, it is important to acknowledge its limitations. One primary limitation of this simulation study is the reliance on synthetic data to evaluate the performance of the multimodal inverse kinematic approach. We have tried to create IMU-based data as realistically as possible by adding various artificial errors to the data. However, we cannot guarantee that the data will be equivalent to real-life sensor data. The influence of corrupted magnetic heading has been implemented by adding a yaw drift error to each virtual sensor. Further magnetic disturbances (e.g. variable misalignment) have not been implemented. Errors stemming from magnetic disturbance are not definitive and therefore difficult to model correctly and cohesively. This remains a limitation of this simulation study. As a result, we cannot yet draw definitive conclusions about the method’s efficacy when applied to real-world measurement data, or about its generalizability. The decision to use virtual sensor data was driven by the main objective of this study: to determine whether single position references can improve kinematic and dynamic analysis outcomes compared with IMU-based results. Additionally, we sought to assess the required accuracy level of spatial data to enhance IMU-based analyses. Our findings indicate that even the least accurate positional data (with errors up to ${3}\,\hbox {cm}$) substantially improve analysis results. Typical IMU errors, such as offset from calibration issues or joint angle drift, were consistently mitigated. This error compensation is notably evident in degrees of freedom with limited ranges of motion, such as hip adduction and hip rotation, as demonstrated in Fig. 2. Specifically, for hip adduction, an offset error correction is particularly pronounced. This error reduction leads to markedly improved dynamic results, as the discrepancies between the reference curve and the IMU-based curve are negligible in the multimodal results. Peak deviations are effectively compensated, as illustrated in Fig. 4.

Further, it is important to note that, despite improvements in dynamic results, residual forces and torques remain evident. In the multimodal approach, these residual forces and torques range between $\sim \pm {70}\,\hbox {N}$ and $\sim {20}\,\hbox {Nm}$, respectively. Although the residual forces fall below the recommended threshold of ${5}{\%}$ of the net external forces, the residual torques exceed the suggested limit of ${1}{\%}$ of the net external torques^20^. Adjustments made to the spatial reference weighting of the base segment (the pelvis) did not result in a reduction of residual forces and torques. Subsequent analysis of the residual forces and torques per gait phase (stance and swing) demonstrate consistent values. As apparent from the coordinate components, the x-y-coordinate values are generally higher than the z-component values, with the exception of residual forces for the IMU-based approach in the swing phase. However, the primary aim of this study was not to introduce a novel method to minimize residuals, but rather to explore whether incorporating multimodal data in an inverse kinematics approach enhances biomechanical analysis outcomes. Both qualitative and quantitative analyses of the dynamic analysis results demonstrated a consistent improvement over IMU-based results due to the inclusion of spatial reference data. Residual forces and torques reflect various errors in the inverse analysis approach, such as model uncertainties or measurement inaccuracies. Minimizing the kinematic error as much as possible is advantageous for reducing overall residuals, as it allows other error sources to be addressed through alternative methods.

One key benefit of our approach is that it does not impose any restrictions on the movement to be analyzed or the dimensionality of the model used. Inverse kinematics methods have already been used to analyze a wide variety of motions using three-dimensional models: e.g. (deep) squat^38,39^, upper body reaching motions^31^, side cut^40,41^ or jump lunge^40^. This distinguishes our inverse kinematics-based approach from trajectory optimization approaches used to process IMU-based data without drift, which have thus far been limited to analyzing two-dimensional gait or running motions. Although gait data was also analyzed in this study, three-dimensional motion data was used. In addition, given that inverse kinematics–based analyses have been successfully applied to a variety of captured movements, we anticipate that our method will generalize well to other motion types. Future work will focus on evaluating the performance of the proposed approach using real-world measurement data across different movement tasks.

Another key advantage of the proposed multimodal inverse kinematics approach is that it is largely independent of the measurement system used. While the method requires specific input modalities – namely, position and orientation data – it is not limited to a particular technology for acquiring these inputs. Recent advances in motion capture have introduced various techniques, including those based on RGB cameras^11–13^, depth cameras^14,15^, and radar^18,19^. Our findings show that input data with relatively low spatial accuracy can still lead to significant improvements in biomechanical analysis outcomes. Consequently, the approach is compatible with a broad range of sensing technologies, highlighting its versatility and practical application.

## Conclusion

In this simulation study using synthetic data, we demonstrated that incorporating spatial information into IMU-based motion data improves both kinematic and dynamic analysis. Future research will evaluate the effectiveness of the multimodal inverse kinematics approach when applied to real-world measurement data of various motions. We plan to use radar-based position data to enhance orientation-based motion capture data using the proposed multimodal approach. In addition, we intend to investigate whether combining a forward dynamic simulation approach with multimodal motion data can further enhance simulation accuracy and reduce residuals more effectively. Future research could also focus on integrating multimodal motion data with comprehensive model individualization methods to help bridge the gap between simulation results and reality.

## Supplementary Information

Supplementary Information.

## Data Availability

The datasets generated during and/or analyzed during the current study are available from the corresponding author on reasonable request.
