# Supplementary material for: Multimodal inverse kinematics significantly improves IMU-based biomechanical analyses
Source: Sci Rep. 2025 Dec 23;15:44420. doi: 10.1038/s41598-025-33021-7 (PMC12738565; doi:10.1038/s41598-025-33021-7)
Supplement: Supplementary file 1 — Supplementary Information. [file 41598_2025_33021_MOESM1_ESM.pdf]

Supplementary Material

Multimodal Inverse Kinematics Significantly Improves IMU-based Biomechanical Analyses

Iris Wechsler, Julian Shanbhag, Niklas Schlechtweg, Martin Vossiek, Anne D. Koelewijn, Sandro Wartzack, and Jörg Miehling

Marker weighting	γ	IMU	Multimodal
9.81	1	8.8369	8.6636
	0.1	8.8369	8.5154
	0.01	8.8369	8.0810
	0.001	8.8369	7.3613
	0.0001	8.7990	5.2298
	0.00001	8.9312	3.5629
	0.000001	8.8369	3.2508
	0.0000001	8.8691	3.7782
	0.00000001	8.7939	3.4471

Supplementary Table S1. Marker weighting and scaling factor α as well as corresponding joint angle RMSEs between reference data and IMU-based and multimodal analysis results.

	Degree of freedom	**IMU**		Multimodal	
		Mean	Std	Mean	Std
Joint translation RMSE	Pelvis Tx	0.10	0.00	0.00	0.00
	Pelvis Ty	0.01	0.00	0.00	0.00
	Pelvis Tz	0.13	0.00	0.03	0.01
Joint angle RMSE	Pelvis Tilt	7.74	1.60	1.51	1.40
	Pelvis List	7.45	1.60	1.89	1.38
	Pelvis Rotation	6.94	2.55	3.16	2.05
	Hip Flexion R	9.01	6.74	2.72	2.14
	Hip Flexion L	7.96	6.72	2.60	2.33
	Hip Adduction R	9.31	5.69	1.97	1.34
	Hip Adduction L	9.97	5.87	1.83	1.30
	Hip Rotation R	10.70	6.28	7.32	4.40
	Hip Rotation L	10.19	5.65	7.03	4.52
	Knee Angle R	8.45	6.81	2.84	2.33
	Knee Angle L	9.77	6.71	2.74	2.40
	Ankle Angle R	9.00	6.81	3.25	2.57
	Ankle Angle L	8.31	6.58	3.34	2.74
Joint force RMSE	Pelvis Tx	42.33	0.84	26.19	13.81
	Pelvis Ty	35.11	1.81	11.78	9.72
	Pelvis Tz	27.35	1.37	40.48	32.58
Joint torque RMSE	Pelvis Tilt	35.91	15.48	10.78	7.27
	Pelvis List	60.04	22.70	17.26	11.00
	Pelvis Rotation	13.83	5.79	4.34	2.90
	Hip Flexion R	21.44	12.36	4.10	1.81
	Hip Flexion L	19.54	11.71	3.60	2.28
	Hip Adduction R	42.51	19.05	3.08	2.42
	Hip Adduction L	34.20	13.79	2.72	2.16
	Hip Rotation R	9.21	4.06	0.81	0.57
	Hip Rotation L	7.22	3.30	0.81	0.57
	Knee Angle R	16.57	5.86	4.28	3.06
	Knee Angle L	14.43	4.67	4.07	2.95
	Ankle Angle R	5.65	2.53	2.49	1.33
	Ankle Angle L	4.77	1.80	2.68	1.68

Supplementary Table S2. Mean and standard deviation values of generalized coordinates (joint translations (m) / joint angles (°)) and joint force (N) / torque (Nm) RMSEs for both approaches in relation to the reference data for each degree of freedom. The translational (N) and rotational degrees of freedom of the pelvis describe the residual forces and torques respectively.

Error type	Error size	Mean difference	CI lower	CI upper	p-value	Sig	N
Bias drift	Small	-5.72	-6.74	-4.71	8.84e-12	*	27
	Medium	-5.55	-6.49	-4.61	3.54e-12	*	27
	Large	-5.48	-6.41	-4.55	3.37e-12	*	27
Gyro noise	Small	-6.34	-7.41	-5.26	3.60e-12	*	27
	Medium	-5.68	-6.43	-4.94	8.13e-15	*	27
	Large	-4.73	-5.66	-3.80	8.24e-11	*	27

**: significant to adjusted alpha value*

Supplementary Table S3. Results of the statistical analysis comparing joint angle RMSEs betwen IMU-based and multimodal solution: mean differences (°), 95% CIs (°), and p-values from t-tests.

Error type	Error size	Mean difference	CI lower	CI upper	p-value	Sig	N
Bias drift	Small	-16.76	-18.94	-14.58	7.89e-15	*	27
	Medium	-17.75	-19.84	-15.65	7.22e-16	*	27
	Large	-17.25	-20.17	-14.32	3.35e-12	*	27
Gyro noise	Small	-15.99	-18.01	-13.97	3.79e-15	*	27
	Medium	-16.16	-18.33	-14.00	1.49e-14	*	27
	Large	-19.60	-22.37	-16.83	5.26e-14	*	27

**: significant to adjusted alpha value*

Supplementary Table S4. Results of the statistical analysis comparing joint torque RMSEs betwen IMU-based and multimodal solution: mean differences (Nm), 95% CIs (Nm), and p-values from t-tests

Marker weighting	ε	Residual	IMU	Multimodal
9.81	0.01	Forces	36.59	17.24
		Torques	34.93	38.00
	0.1	Forces	36.59	13.04
		Torques	34.93	38.71
	10	Forces	36.59	10.67
		Torques	34.93	23.41
	100	Forces	36.59	16.39
		Torques	34.93	38.30

Supplementary Table S5. Pelvis marker weighting and scaling factor ε as well as corresponding residual force and torque RMSEs between reference data and IMU-based and multimodal analysis results.
